# Angiotensin II administration to COVID-19 patients is not advisable

**DOI:** 10.1186/s13054-020-03032-z

**Published:** 2020-06-05

**Authors:** Robert C. Speth

**Affiliations:** 1 Pharmaceutical Sciences in the College of Pharmacy, Nova Southeastern University, Fort Lauderdale, FL USA; 2 Department of Pharmacology and Physiology in the School of Medicine, Georgetown University, Washington, DC, USA

## Background

The COVID-19 pandemic is the worst health crisis to afflict the planet in more than 100 years. It is exacerbated by the lack of effective therapies to prevent the disease. In desperation, many therapies are being devised that are totally devoid of scientific credibility which has led to the US Federal Trade Commission (FTC) to issue warning letters to 45 companies making unsupported claims for COVID-19 cures https://www.ftc.gov/news-events/press-releases/2020/05/ftc-sends-45-more-letters-warning-marketers-stop-making (accessed May 10, 2020). Potentially rational therapies can also be promoted that can arise from misinterpretation or unwarranted extrapolation of preclinical observations [1]. Such therapies have the potential to cause harm to COVID-19 patients. This commentary addresses one such proposed therapeutic approach: administration of angiotensin (Ang) II (Giapreza®) to COVID-19 patients [2]. Hypertension is one of the risk factors associated with the morbidity and mortality of the current COVID-19 pandemic [3], and it is possible that the renin-angiotensin system may exacerbate the severity of COVID-19 [4]. Virtually every medical society promoting cardiovascular health has advised continuing the use of angiotensin-converting enzyme (ACE) inhibitors and angiotensin receptor blockers (ARBs) during the COVID-19 pandemic [5], which contraindicates therapeutic administration of Ang II for treatment of COVID-19 patients.

Overlooking the pathological significance of elevated blood pressure that can be caused by Ang II flies in the face of recommendations of the American Heart Association and more specifically, the findings of the SPRINT trial [6]. While the likelihood of hypotensive shock associated with COVID-19 infection is a concern, there is one report of hypotension associated with COVID-19 infections requiring vasopressor therapy [7], and the vasopressors used were primarily norepinephrine and secondarily vasopressin (Pavan Bhatraju, personal communication, April 10, 2020). Moreover, inferences that individuals with hypertension taking angiotensin-converting enzyme (ACE) inhibitors or angiotensin receptor blockers (ARBs) are at greater risk of injury from the SARS-CoV-2 virus because of ACE2 upregulation [2, 8, 9] is also disconcerting. There is an increasing body of information affirming the value of ACE inhibitor and ARB treatment not only for protection from adverse cardiovascular events, but also for possible therapeutic benefit against COVID-19 morbidity and mortality [4, 10]. The suggestion that ACE inhibitors and ARBs might increase ACE2 expression in human lungs is unsubstantiated [1]. The animal studies of the relationship between ACE inhibitor and ARB administration and ACE2 expression are ambiguous, limited to mRNA expression studies, or limited to cardiac, kidney, or vascular ACE2, and some report no changes in ACE2, as recently reviewed [5]. There are no human studies showing that binding of Ang II to AT_1_ receptors increases ACE2 internalization, thereby downregulating ACE2 in the lungs.

It should be noted that Figure 1 of Busse et al. [2] portrays the renin-angiotensin without including angiotensin I or ACE1. Nor is there any representation of Ang-(1-7) the product of ACE2 metabolism of Ang II in the diagram. Angiotensin II is portrayed as a ligand that binds to ACE2 rather than as a substrate that is rapidly metabolized to Ang-(1-7). The AT_1_ receptor and ACE2 are portrayed as if they were heterodimerized and internalized concurrently, serving as the only means whereby ACE2 is internalized.

Given the similarity between SARS-CoV-1 and SARS-CoV-2, it is not surprising that SARS-CoV-2 downregulates ACE2 [11], as has been shown for SARS-CoV-1, with serious adverse consequences [12]. Thus, any attempts to further downregulate ACE2 with Ang II administration would likely have even more serious adverse consequences.

Inflammation arising from a cytokine storm is one of the major causes of morbidity of SARS-CoV-2 infection, and the ability of Ang II to cause inflammation by activating AT_1_ receptors is well established [13]. To cite just one of many studies, in a mouse lipopolysaccharide (LPS)-induced acute lung injury (ALI) model, exogenous ACE2 reduced pathological injury to the lung and improved lung function [14]. Two mechanisms were demonstrated for this beneficial effect: metabolic inactivation of Ang II and formation of Ang 1-7. The beneficial effects of ACE2 administration were diminished by an Ang 1-7 antagonist and an AT_1_ receptor blocker [14].

## Conclusions

As noted above [5], the American Heart Association, American College of Cardiology, and many other biomedical societies recommend continuing ACE inhibitor and ARB therapy for hypertension. Thus, the deployment of angiotensin II as a vasopressor would be both unsound in patients on ARB therapy and counter to the established antihypertensive and putative therapeutic effects of ACE inhibitors and ARBs for COVID-19. Of note, as of May 10, 2020, there were 9 trials registered on clinicaltrials.gov to assess the therapeutic benefits of ARBs for treating COVID-19 infections, two for Ang 1-7, and none for Ang II.

## Data Availability

Supporting data derives from publications cited in the commentary.
